# Awareness of energy drink intake guidelines and associated consumption practices: a cross-sectional study

**DOI:** 10.1186/s12889-015-2685-2

**Published:** 2016-01-05

**Authors:** Amy Peacock, Nicolas Droste, Amy Pennay, Peter Miller, Dan I. Lubman, Raimondo Bruno

**Affiliations:** 1 School of Medicine (Psychology), University of Tasmania, Hobart, Australia; 2 School of Psychology, Deakin University, Geelong, Australia; 3 Turning Point, Eastern Health, Melbourne, Australia and Centre for Alcohol Policy Research, La Trobe University, Melbourne, Australia; 4 Turning Point, Eastern Health, Melbourne, Australia and Eastern Health Clinical School, Monash University, Melbourne, Australia; 5 National Drug and Alcohol Research Centre, University of New South Wales, Sydney, NSW Australia

**Keywords:** Energy drink, Caffeine, Policy, Labelling, Regulation, Warning

## Abstract

**Background:**

Despite concern regarding harms of energy drink (ED) consumption, no research has been conducted to determine awareness and compliance with ED intake guidelines displayed on product packaging in Australia (a novel approach internationally).

**Methods:**

A convenience sample of 1922 people completed an online survey. Participants reported their knowledge of maximum recommended daily ED intake according to Australian guidelines.

**Results:**

Guideline awareness was reported by 38, 23 and 19 % of past year consumers, lifetime, and non-consumers, respectively. Amongst past year consumers, ‘*accurate estimators*’ reported greater ED intake and were more likely to exceed intake guidelines and consume alcohol mixed with ED (AmED). After controlling for demographics and frequency of use, guideline awareness predicted increased likelihood of exceeding guidelines in ED sessions, but was not associated with exceeding ED guidelines in AmED sessions.

**Conclusions:**

Australia is considered to have the most stringent regulatory approach to EDs internationally. However, advisory statements are not associated with greater awareness and compliance with intake guidelines. Failure to comply with standards for efficacious product labelling, and absence of broader education regarding guidelines, needs to be addressed.

**Electronic supplementary material:**

The online version of this article (doi:10.1186/s12889-015-2685-2) contains supplementary material, which is available to authorized users.

## Background

Energy drinks (EDs) are beverages advertised as enhancing energy, alertness and performance. EDs have increased in popularity in the last decade: half of an Australian population-based young adult sample reported monthly or more frequent use [1], with research suggesting that the average age of onset for ED use is 10 years [2]. Data from Australian and US poison information call centres and emergency departments show increasing numbers of ED-related exposure cases in the last decade [3–6]. Consumers report negative stimulant effects of ED consumption, including headaches, heart palpitations, agitation, and sleeping difficulties [7–9], with more serious side-effects evident in clinical data, particularly after excessive consumption or in the presence of an existing medical condition [10–12]. Consequently, researchers and health professionals have expressed concern that these beverages may cause harm when consumed in excess [13].

The negative effects of EDs are attributed to the primary psychoactive ingredient, caffeine, as the typical symptom profile matches that evident following excessive caffeine intake [14]. Australia and Canada have requirements that ED product labelling must specify the caffeine content in the beverage [15, 16]; the US and European Union only require such labelling if classified as a ‘beverage’ or if content exceeds 150 mg/L, respectively [17, 18]. Australia and Canada additionally require an intake advisory statement (e.g., “Consume no more than [quantity (as cans, bottles or mL)] per day”). In Australia, the maximum recommended daily intake is 500 mL ED (160 mg caffeine) per day (exceedance of this guideline hereafter referred to as ‘excess intake’). As of August 2008, Food Standard Australia Zealand required that this guideline be conveyed to consumers via the advisory statement printed on the beverage container. However, there are no legal requirements as to the wording, location, size, and visibility of this statement, and this advisory statement has been described as confusing, of low visibility, and often worded to encourage, rather than discourage, excess consumption [19, 20].

Health advisory statements are targeted at “information-sensitive” consumers who are able and willing to attend to, comprehend, recall, and act on the message. However, attention is impacted by bottom-up factors (e.g., characteristics of the advisory statement) as well as top-down factors [e.g., motivations for use; 21]. Even if the message is processed, consumers will engage in lay epidemiology to justify their preferred consumption practices, and will act upon existing beliefs, experiences or attitudes which alter their interpretation of the health message [21–23]. Prior research assessing advisory statements for other products provide mixed support in regards to awareness and compliance. The majority of the population is well aware of the harmful effects of smoking because of a range of evidence-based measures, including clear front of pack labelling, with some evidence of efficacy in promoting smoking cessation and preventing smoking initiation [24, 25]. The impact of health warning labels is dependent upon their location, size and design, with poorer recall of obscure text-only warnings which appear on the side or back of packaging [24]. Indeed, alcohol labelling continues to achieve poor levels of recall [26–28], similar to that observed for obscure text-only tobacco labelling [24], due to less-than-optimal size, positioning, and design of labelling. Better recall of labelling has been associated with heavier alcohol consumption [26, 29], presumably due to greater exposure over time to alcohol packaging.

Despite increasing concern regarding the potential harms of excess ED consumption [13] and evidence for varying efficacy of labelling advisory statements, no research has been conducted to date to determine consumer awareness of, or behavioural compliance with, ED intake guidelines as specified via the labelling advisory statement. Given increasing popularity of alcohol mixed with energy drink (AmED) consumption and evidence of increased likelihood of excess ED consumption when consuming AmED [30, 31], it is crucial that any attempts to capture awareness and behavioural compliance also takes into account consumption practices when EDs are consumed independently and in combination with alcohol. Consequently, the aims of the present exploratory study were to assess:

1. Awareness of ED intake guideline (according to product labelling) among an Australian community sample;
2. Compliance with guidelines according to consumers’ awareness of guidelines;
3. Demographic profile and AmED consumption practices of consumers according to guidelines awareness; and
4. Whether awareness of guidelines is independently associated with actual ED consumption (in ED and AmED drinking sessions) after controlling for other covariates for consumption, including frequency of use.

## Methods

### Participants and procedure

A convenience sample of 2953 people aged 16 years or older residing in New South Wales, Australia, completed an online survey between December 2012 and February 2013. An online advertising campaign was adopted to deliberately and non-randomly capture consumers of ED in New South Wales, including advertising on social media websites and internet forums, media reports, and email snowballing amongst professional and university-based networks. Participants were invited to complete the survey regardless of their history of ED use. Participants provided informed consent and survey completion took 15–30 min. After submitting their responses, participants could redirect to a secure webpage and enter a prize draw to win one of ten Apple iPads. Participant Internet Protocol (IP) addresses were collected and stored independently of survey responses to ensure each participant was unique. Ethics approval was granted by the Deakin University Human Ethics Advisory Group (#2012-257).

### Measures

Participants were informed throughout the survey that an ED was defined as “any caffeinated drink which is advertised primarily as providing benefits to physical and mental performance, endurance, concentration and/or stamina”. This definition was accompanied by images of common products in Australia, and quantification of a standard ED unit (one 60 mL ED shot = 1 standard ED; one 250 mL ED beverage = 1 standard ED; one 500 mL ED beverage = 2 standard EDs). This quantification was based on standard beverage packaging and composition of products (80 mg of caffeine per 250 mL beverage, the maximum concentration permissible under current regulation).

In addition to items pertaining to demographics (age, sex, currently employed, currently studying for a tertiary qualification, and completed a tertiary qualification), participants were asked “According to Australian guidelines, what is the maximum recommended amount of standard EDs that should be consumed per day?”.

Participants were also asked about their average daily caffeine intake (mgs) by reporting typical daily intake of caffeinated products (excluding EDs). The caffeine content of foods and beverages was based on the Australia New Zealand Food Standards nutrient database [32] or product packaging. Participants were also asked about lifetime and past 12 month ED use and AmED use. Past 12 month consumers were asked about frequency of use (‘less than monthly’, ‘2–4 times a month’, ‘2–3 times a week’, and ‘four or more times a week’), and ED intake in typical and maximum ED and AmED sessions in the past 12 months. Alcohol intake estimates in typical and maximum AmED sessions were also obtained from those participants who reported using AmED. Based on the Australian guidelines, the maximum recommended daily ED intake was defined as two standard ED units (2 x 250 mL ED) [15] and maximum alcohol intake to reduce risk of alcohol-related injury was defined as four standard drinks [33].

### Availability of data and measures

The survey instrument and data can be made available on request of the corresponding author.

### Analyses

The inclusion rate following data cleaning was 65.1 % (*n =* 1922). Cases were removed if participants were not a NSW resident or if there were missing data for items deemed critical to the research question (i.e., age, alcohol/ED consumption intake and frequency, and guideline awareness) or survey progress logic. Participants were grouped according to ED consumer status: ‘*past year consumer’* (used in the past 12 months), ‘*lifetime consumer*’ (used in lifetime but not in the last 12 months) and ‘*non-consumer’* (never consumed ED in their lifetime); and by awareness of guidelines: ‘*accurate estimators’* (2 standard EDs), ‘*under-estimators’* (1 standard ED), ‘*over-estimators’* (≥3 standard EDs) and ‘*unaware consumers’* (‘don’t know’). Binary variables were also created for ED intake (‘within guidelines ≤2 ED’ versus ‘exceeding guidelines ≥3 ED’) and alcohol intake (‘within guidelines ≤4 standard alcoholic drinks’ versus ‘≥5 standard alcoholic drinks’).

Analyses were conducted in SPSS Statistics Version 21 (IBM, Somers, NY) unless otherwise indicated. Descriptive statistics comprised percentages and 95 % confidence intervals (95 % CI) for categorical outcomes and mean and standard deviation for continuous outcomes (Aim 1). Odds ratios were calculated to compare year ED consumers based on guideline awareness for DE consumption, socio-demographic and AmED consumption outcomes (Aim 2 and 3) using Comprehensive Meta-Analysis Version 2 (Biostat, Englewood, NJ).

In order to determine whether guideline awareness is independently associated with actual consumption (Aim 4), three-step hierarchical binary logistic regressions were conducted; the dependent variable was whether past year ED consumers had exceeded ED intake guidelines in typical and maximum ED sessions. As awareness of ED guidelines is intended to result in behavioural compliance with guidelines, ED guideline awareness (‘*under-estimators/over-estimators/unaware consumers*’ (hereafter referred to as ‘*uninformed consumers*’) versus ‘*accurate estimators’*) was entered at Step 1. Age, sex, employment, educational qualification and current studying status were entered at Step 2 based on research showing that ED users are generally male and younger; employment and educational attainment were also included in the model given contradictory findings regarding their association with ED consumption [8, 34, 35]. Frequency of ED use was entered at Step 3 based on literature showing that level of exposure to advisory statements impacts awareness and behavioural compliance [22]. As a proxy for overall ED exposure, a variable was created to reflect the highest frequency of use for ED for each participant (i.e., selected highest frequency of use for ED or AmED).

Four-step hierarchical binary logistic regressions were conducted to determine predictors of excess intake in typical and maximum AmED sessions. Models were as outlined above, with the exception that the binary variable for exceeding alcohol intake guideline (‘within guidelines: ≤4 standard alcoholic drinks’ versus ‘≥5 standard alcoholic drinks’) in typical and maximum AmED session was included in Step 4. Across all analyses, significance levels were maintained at *p* < .05.

## Results

### Sample characteristics

The sample predominantly comprised male (58 %, 95 % CI 55–61) young adults (*M* = 24.4, *SD* = 6.7). Half the sample had acquired a diploma or tertiary qualification (48 %, 95 % CI 45–50), nearly four-fifths were currently studying for a diploma or tertiary qualification (78 %, 95 % CI 77–81) (60 %, 95 % CI 57–63) and approximately half were currently employed (48 %, 95 % CI 45–50). Of the total sample, 80 % (95 % CI 78–81; *n* = 1530) had consumed an ED in their lifetime, and 59 % (95 % CI 57–61, *n* = 1130) had consumed an ED in the past year.

### Awareness of guidelines

Analysis of guideline awareness by ED consumer status showed that approximately two-fifths (38 %) of past year consumers accurately reported the maximum recommended daily ED intake. Almost one-third (31 %) underestimated and over one-quarter (27 %) were unsure of the guideline, respectively; only a minority (5 %) overestimated the recommended maximum daily intake (Table 1). One-half of lifetime consumers (51 %) and non-consumers (56 %) reported being unaware of the guideline, with around one-fifth (23 and 19 %, respectively) correctly reporting the maximum recommended daily ED intake.

**Table 1:** Awareness of guidelines according to history of energy drink use

Awareness of guidelines	Non-consumer	Lifetime consumers	Past year consumers
	*n* = 387 % (95 % CI)	*n* = 400 % (95 % CI)	*n* = 1130 % (95 % CI)
Underestimated	23 (19–28)	24 (20–28)	31 (28–34)
Correct	19 (16–23)	23 (19–27)	38 (35–41)
Overestimated	2 (1–4)	3 (2–5)	5 (4–6)
Don’t know	56 (51–61)	51 (46–56)	27 (24–29)

Five participants who had not used ED in the past year did not identify whether they had consumed ED in their lifetime

### ED Use according to guideline awareness

Comparison of ED intake (without alcohol) of past year consumers on the basis of their guidelines awareness showed that ‘*under-estimators*’, ‘*over-estimators*’ and ‘*unaware consumers*’ were less likely to report ED use every two weeks or more frequent than ‘*accurate estimators*’ (39, 30, and 26 % versus 46 %, respectively). These three groups were also less likely to report exceeding the guidelines on a monthly or more frequent basis as compared to the ‘*accurate estimators*’ (11, 10, and 7 % versus 19 %, respectively), although the comparison with the ‘*over-estimators*’ group did not reach statistical significance.

‘*Under-estimators*’ and ‘*unaware consumers*’ reported significantly lower ED intake in typical and maximum ED sessions than ‘*accurate estimators*’, with significantly fewer consumers in the former two groups exceeding the ED intake guidelines in these sessions (Table 2). Most notably, 43 % of ‘*accurate estimators*’ reported exceeding the guidelines in their maximum sessions while almost half that percentage (27 and 24 %) reported this behaviour in the ‘*under-estimators*’ and ‘*unaware consumers*’ groups. Consumption patterns for ‘*over-estimators*’ and ‘*accurate estimators*’ were generally similar (although low statistical power should be noted here given the small sample size).

**Table 2:** Energy drink consumption (without alcohol) of past year consumers according to awareness of guidelines

	A. accurate estimators	B. under-estimators	C. over-estimators	D. unaware consumers	B. vs A. (ref) OR	C. vs A. (ref) OR	D. vs A. (ref) OR
	*n* = 398	*n* = 324	*n* = 50	*n* = 274	(95 % CI)	(95 % CI)	(95 % CI)
% ED use every two weeks +	46 (41–51)	39 (33–44)	30 (19–44)	26 (21–32)	**0.75 (0.55–1.00),** ***p*** **= .054**	**0.51 (0.27–0.96),** ***p*** **= .037**	**0.42 (0.30–0.59),** ***p*** **< .001**
% Monthly or more frequent intake in excess of guidelines	19 (15–23)	11 (8–15)	10 (4–21)	7 (5–11)	**0.54 (0.35–0.83),** ***p*** **= .005**	0.48 (0.18–1.24), *p* = .130	**0.34 (0.20–0.57),** ***p*** **< .001**
Typical ED intake (M, SD)	1.6 (1.1)	1.4 (0.7)	1.7 (1.7)	1.4 (1.0)	**0.74 (0.62–0.89),** ***p*** **= .002**	1.04 (0.83–1.31), *p* = .724	**0.82 (0.69–0.98),** ***p*** **= .027**
% Typical intake exceeds ED guidelines	8 (6–10)	11 (8–15)	6 (4–9)	9 (4–20)	**0.49 (0.27–0.87),** ***p*** **= .016**	0.75 (0.26–2.21), *p* = .605	**0.54 (0.30–0.99),** ***p*** **= .046**
Maximum ED intake (M, SD)	2.9 (2.3)	2.3 (1.7)	2.9 (3.7)	2.1 (2.0)	**0.87 (0.80–0.95),** ***p*** **= .001**	1.01 (0.90–1.12), *p* = .921	**0.81 (0.74–0.90),** ***p*** **< .001**
% Maximum intake exceeds ED guidelines	43 (38–48)	27 (22–32)	36 (23–50)	24 (20–30)	**0.50 (0.36–0.69),** ***p*** **< .001**	0.74 (0.39–1.41), *p* = .364	**0.44 (0.31–0.62),** ***p*** **< .001**

Twenty-five participants did not specify whether they had consumed EDs independently (*n* = 1105). Those values bolded indicate statistical significance (*p* < .050). Energy drink: energy drink; *OR* odds ratio, *95 % CI* 95 % confidence interval

### Correlates of guideline awareness

The following analyses comprised comparison of ‘*accurate estimators*’ versus the ‘*uninformed consumers*’ (referent category; ‘*under-estimators*’, ‘*over-estimators’* and ‘*unaware consumers*’ combined); descriptive data for all four groups are available in Additional file 1. Whilst the groups were similar in regards to employment, education and typical caffeine intake, ‘*accurate estimators*’ were significantly younger and had greater odds of being female than ‘*uninformed consumers*’ (Table 3).

**Table 3:** Demographic profile and AmED consumption practices of past year ED consumers according to guideline awareness

	N	Total sample	A. uninformed consumers *n* = 703	B. accurate estimators *n* = 427	B vs A (ref) OR (95 % CI)
Demographics and other drug use					
Age (M, SD)	1130	24.4 (6.7)	25.0 (7.4)	23.5 (5.2)	**1.49 (1.20–1.86),** ***p*** **< .001**
% Male	1124	58 (55–61)	61 (57–65)	53 (48–58)	**0.73 (0.57–0.93),** ***p*** **= .011**
% Employed	1129	60 (57–63)	62 (58–65)	57 (53–62)	0.84 (0.66–1.07), *p* = .152
% Completed tertiary qualification	1123	48 (45–50)	49 (45–52)	46 (41–50)	0.89 (0.70–1.13), *p* = .342
% Currently studying tertiary qualification	1130	79 (77–81)	78 (75–81)	80 (76–84)	1.13 (0.84–1.52), *p* = .434
Daily caffeine intake (excluding ED; M, SD)	1136	205.5 (190.8)	162.5 (183.1)	200.5 (199.5)	1.06 (0.85–1.32), *p* = .582
Independent ED Use (past year)	1105	95 (93–96)	94 (92–96)	95 (93–97)	1.29 (0.74–2.26), *p* = .367
		*n* = 1046	*n* = 648	*n* = 398	
Amongst consumers:					
% ED use every two weeks +	1046	36 (33–39)	33 (29–36)	46 (41–51)	**1.73 (1.34–2.24),** ***p*** **< .001**
Typical ED intake (M, SD)	976	1.5 (1.0)	1.4 (1.1)	1.6 (1.1)	**1.47 (1.16–2.10),** ***p*** **= .001**
% Typical intake exceeds ED guidelines	976	8 (6–10)	6 (5–8)	11 (8–15)	**1.89 (1.19–3.01),** ***p*** **= .007**
% Monthly + exceed ED guideline use	1041	13 (11–15)	10 (7–12)	19 (15–23)	**2.23 (1.55–3.20),** ***p*** **< .001**
Maximum ED intake (M, SD)	995	2.5 (2.2)	2.3 (2.0)	2.9 (2.3)	**1.67 (1.32–2.10),** ***p*** **< .001**
% Maximum intake exceeds ED guidelines	995	33 (30–36)	27 (23–30)	43 (38–48)	**2.06 (1.57–2.70), ** ***p*** ** < .001**
AmED Use (past year)	1130	64 (62–67)	61 (58–65)	69 (65–74)	**1.43 (1.10–1.84),** ***p*** **= .006**
		*n* = 727	*n* = 431	*n* = 296	
Amongst consumers:					
% AmED use every 2 weeks +	722	19 (16–22)	15 (12–19)	25 (20–30)	**1.78 (1.22–2.58),** ***p*** **= .002**
Typical ED intake (M, SD)	713	3.0 (2.6)	3.1 (2.8)	3.0 (2.1)	1.05 (0.80–1.38), *p* = .721
% Typical ED intake exceeds guideline	713	42 (38–46)	41 (36–45)	44 (39–50)	1.16 (0.86–1.57), *p* = .336
Typical alcohol intake (M, SD)	719	5.9 (4.1)	5.5 (3.9)	6.5 (4.4)	**1.52 (1.16–1.99),** ***p*** **= .002**
% Typical alcohol intake exceeds guideline	719	54 (51–58)	51 (46–55)	59 (53–65)	**1.41 (1.04–1.90),** ***p*** **= .026**
Maximum ED intake (M, SD)	710	3.8 (3.0)	3.7 (3.0)	4.0 (2.9)	1.21 (0.92–1.59), *p* = .172
% Maximum ED intake exceeds guideline	710	57 (53–60)	55 (50–60)	60 (54–65)	1.21 (0.90–1.64), *p* = .211
Maximum alcohol intake (M, SD)	712	8.3 (6.7)	7.7 (6.7)	9.0 (6.6)	*3.71 (0.9–15.02), p = .066*
% Maximum alcohol intake exceeds guideline	712	64 (60–67)	59 (54–64)	71 (65–76)	**1.69 (1.23–2.33),** ***p*** **= .001**

Those values bolded indicate statistical significance (*p* < .050) and those italicised indicated a trend towards statistical significance (*p* < .100). *ED* energy drink, *AmED* alcohol mixed with energy drink, *M* mean, *SD* standard deviation

‘*Accurate estimators*’ had increased odds of reporting past year AmED use relative to ‘*uninformed consumers’*. Amongst those reporting past year AmED use, ‘*accurate estimators*’ had almost two-fold increased odds of reporting AmED use every two weeks or more frequent, and they reported greater alcohol intake in typical and maximum sessions as compared to ‘*uninformed consumers’*. ED intake in AmED sessions was generally similar across the groups, with approximately two-fifths (41 and 44 %) exceeding the guidelines in their typical AmED session and three-fifths (55 and 60 %) exceeding the guidelines in their maximum AmED session for ‘*uninformed consumers’* and ‘*accurate estimators*’, respectively.

### Guideline awareness as a predictor of exceeding the ED guidelines

#### Exceeding ED guidelines in typical and maximum independent ED sessions

Three-step hierarchical binary logistic regression analyses showed that at Step 1, being an *‘accurate estimator’* was significantly associated with exceeding guidelines in typical and maximum independent ED drinking sessions, accounting for a small amount of variance (2 and 4 %, respectively; Table 4). After controlling for demographics (Step 2), there remained two-fold increased odds of exceeding the guidelines in both session types for *‘accurate estimators’* and those who were male (controlling for age, employment, and education), although these models still accounted for only a small amount of variance in consumption (5 and 9 %, respectively). Controlling for frequency of consumption in these models (Step 3) reduced the magnitude of effect of regulation estimation, with there being a 50 % increase in odds of exceeding guidelines in both session types, and dropping to trend level effects for typical sessions. However, the inclusion of frequency of ED consumption in these models substantially increased the explained variance in consumption (17 and 33 % for typical and maximum ED sessions, respectively). At Step 3, being male and consuming EDs more than weekly was significantly associated with exceeding the guidelines in typical ED drinking sessions, with a trend towards *‘accurate estimators’* reporting a greater likelihood of exceeding the guidelines.

**Table 4:** Predictors of exceeding ED guidelines in typical and maximum ED sessions and AmED sessions

	Exceed guidelines: typical ED session (*n* = 962)			Exceed guidelines: maximum ED session (*n* = 981)			Exceed guidelines: typical AmED session (*n* = 704)			Exceed guidelines: maximum AmED session (*n* = 694)		
Model	*b*	*p*	OR (95 % CI)	*b*	*p*	OR (95 % CI)	*b*	*p*	OR (95 % CI)	*b*	*p*	OR (95 % CI)
Step 1	*R* ^*2*^ ***=*** .017			*R* ^*2*^ ***=*** .040			*R* ^*2*^ ***=*** .002			*R* ^*2*^ ***=*** .003		
Accurate estimator	**0.63**	**.008**	**1.88 (1.18–3.01)**	**0.74**	**<.001**	**2.10 (1.60–2.76)**	0.15	.339	1.16 (0.86–1.57)	0.21	.183	1.23 (0.91–1.67)
Step 2	*R* ^*2*^ ***=*** .045			*R* ^*2*^ ***=*** .091			*R* ^*2*^ ***=*** .029			*R* ^*2*^ ***=*** .028		
Accurate estimator	**0.62**	**.011**	**1.86 (1.15–3.00)**	**0.69**	**<.001**	**1.99 (1.50–2.63)**	0.10	.541	1.10 (0.81–1.50)	0.18	.270	1.19 (0.87–1.63)
Age	0.03	.158	1.03 (0.99–1.07)	−0.01	.267	0.99 (0.96–1.01)	−0.02	.219	0.98 (0.95–1.01)	0.00	.937	1.00 (0.97–1.03)
Male	**0.72**	**.003**	**2.05 (1.27–3.34)**	**0.77**	**<.001**	**2.17 (1.64–2.86)**	**0.52**	**.001**	**1.69 (1.24–2.29)**	**0.40**	**.011**	**1.50 (1.10–2.04)**
Employed	−0.01	.957	1.01 (0.61–1.68)	−0.05	.718	0.95 (0.71–1.27)	0.19	.247	1.21 (0.88–1.67)	**0.32**	**.049**	**1.38 (1.00–1.91)**
Completed tertiary	−0.27	.334	0.76 (0.44–1.32)	−0.17	.295	0.84 (0.61–1.16)	−0.02	.912	0.98 (0.68–1.40)	−0.19	.292	0.83 (0.58–1.18)
Studying tertiary	0.19	.569	1.21 (0.63–2.32)	0.18	.336	1.20 (0.83–1.75)	0.12	.580	1.12 (0.74–1.73)	0.21	.343	1.23 (0.80–1.87)
Step 3	*R* ^*2*^ ***=*** .116			*R* ^*2*^ ***=*** .329			*R* ^*2*^ ***=*** .073			*R* ^*2*^ ***=*** .089		
Accurate estimator	0.41	.109	1.50 (0.91–2.26)	**0.43**	**.007**	**1.54 (1.12–2.12)**	0.01	.936	1.01 (0.74–1.39)	0.08	.640	1.08 (0.78–1.49)
Age	0.03	.192	1.03 (0.99–1.07)	−0.02	.283	0.99 (0.96–1.01)	−0.02	.320	0.98 (0.95–1.02)	0.01	.715	1.01 (0.98–1.04)
Male	**0.62**	**.014**	**1.86 (1.13–3.05)**	**0.64**	**<.001**	**1.89 (1.38–2.59)**	**0.40**	**.013**	**1.49 (1.09–2.05)**	0.24	.143	1.27 (0.92–1.76)
Employed	−0.05	.852	0.95 (0.57–1.60)	−0.21	.204	0.81 (0.58–1.12)	0.11	.517	1.12 (0.80–1.55)	0.24	.155	1.27 (0.91–1.77)
Completed tertiary	−0.29	.314	0.75 (0.43–1.31)	−0.21	.254	0.81 (0.57–1.16)	0.10	.956	1.01 (0.70–1.45)	−0.16	.409	0.86 (0.59–1.24)
Studying tertiary	0.22	.517	1.25 (0.64–2.44)	0.21	.338	1.23 (0.81–1.88)	0.12	.615	1.12 (0.72–1.73)	0.19	.382	1.21 (0.79–1.87)
Frequency of use												
Monthly or less (ref)	-	-	-	-	-	-	-	-	-	**-**	**-**	**-**
Every two weeks-weekly	0.15	.658	1.17 (0.59–2.29)	**1.18**	**<.001**	**3.25 (2.29–4.62)**	**0.77**	**<.001**	**2.16 (1.49–3.13)**	**0.89**	**<.001**	**2.43 (1.67–3.54)**
More than weekly	**1.49**	**<.001**	**4.44 (2.56–7.73)**	**2.80**	**<.001**	**16.39 (10.58–25.40)**	**0.82**	**<.001**	**2.27 (1.47–3.51)**	**1.02**	**<.001**	**2.76 (1.75–4.35)**
Step 4	-			-			*R* ^*2*^ ***=*** .153			*R* ^*2*^ ***=*** .223		
Accurate estimator	-	-	-	-	-	-	−0.06	.722	0.94 (0.68–1.31)	−0.07	.695	0.93 (0.66–1.32)
Age	-	-	-	-	-	-	−0.01	.598	0.99 (0.96–1.02)	0.02	.220	1.02 (0.99–1.06)
Male	-	-	-	-	-	-	0.20	.228	1.23 (0.88–1.71)	0.03	.855	1.03 (0.73–1.46)
Employed	-	-	-	-	-	-	0.10	.581	1.10 (0.78–1.55)	0.28	.127	1.32 (0.93–1.89)
Completed tertiary	-	-	-	-	-	-	−0.04	.854	0.97 (0.66–1.41)	−0.23	.243	0.79 (0.54–1.17)
Studying tertiary	-	-	-	-	-	-	0.05	.829	1.05 (0.67–1.64)	0.16	.492	1.18 (0.74–1.87)
Frequency of use	-	-	-	-	-	-	-	-	-	-	-	-
Monthly or less (ref)	-	-	-	-	-	-	-	-	-	**-**	**-**	**-**
Every two weeks-weekly	-	-	-	-	-	-	**0.78**	**<.001**	**2.19 (1.50–3.19)**	**0.92**	**<.001**	**2.50 (1.68–3.73)**
More than weekly	-	-	-	-	-	-	**0.82**	**<.001**	**2.26 (1.45–3.52)**	**1.01**	**<.001**	**2.75 (1.70–4.46)**
Exceed alcohol guidelines typical AmED sessions	-	-	-	-	-	-	**1.12**	**<.001**	**3.05 (2.19–4.25)**	-	-	-
Exceed alcohol guidelines maximum AmED sessions	-	-	-	-	-	-	-	-	-	**1.53**	**<.001**	**4.63 (3.26–6.58)**

This table presents the results of multivariate hierarchical binary logistic regression analyses. The dependent variable is whether the past year ED consumer reported exceeding ED maximum recommended daily intake guidelines (≥3 standard 250 mL EDs) in typical and maximum ED and AmED drinking sessions in the preceding 12 months. Step 1: awareness of ED guidelines (‘uninformed consumer’ versus ‘accurate estimator’); Step 2: sex, age, employment, completed tertiary qualification, and studying for tertiary qualification; Step 3: frequency of ED use (‘fortnightly-weekly’ and ‘more than weekly’ versus ‘monthly or less); Step 4 (AmED models only): exceeding alcohol intake guidelines (≥5 standard alcoholic drinks) in typical and maximum AmED sessions. Those values bolded indicate statistical significance (*p* < .050) and those italicised indicated a trend towards statistical significance (*p* < .100). ED: energy drink; AmED: alcohol mixed with energy drink OR: odds ratio. 95 % CI: 95 % confidence interval

Similar findings were evident for maximum ED sessions; being an *‘accurate estimator’,* male, and reporting ED use every two weeks-to-weekly, or ED use more than weekly were independent predictors of exceeding the guidelines in these sessions.

#### Exceeding ED guidelines in typical and maximum AmED sessions

Four-step hierarchical binary logistic regression analyses showed that at Step 1, being an *‘accurate estimator’* was not significantly associated with exceeding ED intake guidelines in typical and maximum AmED ED drinking sessions. This absence of any relationship between guideline awareness and behaviour remained even after for controlling for demographics (Step 2), frequency of ED use (Step 3) and AmED use (Step 4).

## Discussion

The present study demonstrated that approximately two-fifths of past year consumers are aware of ED intake guidelines. The literature on alcohol and tobacco labelling suggest that vividness-enhancing characteristics (large-print, simple message, graphic images) and location (isolated and occupying a large area of the package) must be maximised to garner attention [36, 37]. However, in Australia there are no regulatory requirements for presentation of ED advisory statements beyond the inclusion of a text-based warning on packages. This is somewhat surprising given the wealth of literature outlining basic principles and guidelines for efficacious design of such messages [38]. Given the current findings, there is sufficient evidence to suggest that current regulation could be amended to more stringently regulate presentation of the advisory statement, maximising the opportunity for consumers to become aware of guidelines.

However, awareness of advisory statements does not necessarily translate into behavioural compliance. Indeed, this study demonstrated the inverse: that those who were aware of the intake guidelines were more likely to exceed the guidelines as compared to those who were inaccurate or were unaware. Even after controlling for demographics and frequency of use, guideline awareness predicted increased likelihood of exceeding guidelines in typical and maximum ED sessions (but not significantly so), and was not associated with exceeding guidelines in typical and maximum AmED sessions. These findings are not surprising given a wealth of literature showing a ‘boomerang effect’ of public health interventions, whereby effects contrary to those intended are evident [39].

The simplest explanation for these findings is that regular ED consumers and those who consume multiple EDs on a night out have more opportunity to observe the guideline labels than those who use EDs less frequently and in smaller amounts. Greater opportunity to read the guidelines may therefore result in better knowledge of the guidelines. Another explanation for these outcomes is that ‘*accurate estimators*’ might experience psychological reactance, whereby oppositional attitudes and behaviours are created in response to perceived threats to personal choice [39]. The results showed that ‘*accurate estimators*’ consumed ED more frequently, and greater frequency of use was shown to be a stronger independent predictor of exceeding the guidelines even after controlling for guideline awareness. Greater familiarity with the product has been linked with better recall but less attention to, and behavioural compliance with, advisory statements [22]. Thus, it may be that increased personal experience with the product allows consumers to develop greater tolerance, as well as independently judge the safety of the product, thus increasing the chance of exceeding the guidelines. This form of lay epidemiology helps to explain consumer justification of contrarian health behaviours in the face of public health messages, such as the use of illicit opioids and binge alcohol consumption [23, 40] In this manner, ‘*accurate estimators’* who report exceeding guidelines are acting as calculating consumers, whereby the risk of negative health outcomes is weighed against personal and peer experiences, the intended function of the drink, habitual use, physiological dependence, motives for consumption, and personal beliefs.

Given the cross-sectional nature of this study, we cannot directly infer that awareness of guidelines influences ED intake. However, the efficacy of these advisory statements as a stand-alone approach to harm reduction for EDs must be considered. Some researchers argue that an advisory statement can be deemed successful if it informs consumers of potential risks even if behavioural compliance is not forthcoming, whilst others advocate for measurable increases in behavioural compliance as the indicator of efficacy (see Ringold, 2002; [39]). However, the current study shows that: 1) there is low awareness of the guidelines amongst ED consumers, and 2) in the main, awareness of guidelines does not necessarily translate to healthier decisions in regards to ED intake. The basic theoretical underpinnings of risk communication is that in order to make decisions about the risks associated with a particular product, consumers need to understand the type and likelihood of negative outcomes, what factors alter susceptibility to these outcomes, and how they might avoid harm [41]. That is, people need to be informed of the severity and probability of harm, and difficulty of avoiding harm [41]. Consequently, it is not surprising that for most other potentially hazardous products, advisory statements are included as part of an integrated multi-faceted broader campaign seeking to educate and inform consumers, integrated as part of school curriculum regarding alcohol and other drug use and associated harms. Including only an advisory statement means that there is no way to ensure consumers understand the warning, and determine whether they perceive it as accurate. The current findings speak to the importance of undertaking such an approach for ED consumption to ensure that the advisory statements are placed in a broader context of knowledge and understanding of adverse outcomes and harm reduction strategies.

This study was the first to systematically test awareness of ED guidelines amongst a large sample of consumers and non-consumers, providing critical insight into the need for better policy responses in Australia and internationally. Direct comparison of ‘over-estimators’ with other guideline awareness group was not possible due to small group size resulting in low statistical power. It is important to note that we did not explicitly assess whether participants were aware of the guidelines from the product packaging. However, we can confidently assume that this knowledge was derived from the advisory statement on ED packaging, as we know of no other public health initiative in Australia to promote these guidelines. It also should be noted that data were self-reported and thus subject to potential bias, although a web-based survey was used to allow independent completion, and non-identifying information was collected to assure anonymity. We also advocate for research which assesses guideline awareness in the broader community; the sample was not representative as participants were self-selected as part of a strategic recruitment plan to target AmED consumers. For example, 58 % of the current sample were male and median age was 24, whereas in NSW in June 2013 the sex distribution was near-equal and median age was 38 years [42]. Critically, we believe the next step is to undertake longitudinal research using consumers in the community to explore how an individual consumer transitions from lack of knowledge regarding the guidelines to a state of awareness and subsequently to behavioural compliance.

## Conclusions

This study has shown that less than two-fifths of all ED consumers in our Australian sample were aware of recommended maximum daily intake guidelines. Further, the current findings suggest that awareness of guidelines explains little of the variance in ED intake, and may actually be associated with greater likelihood of excess intake. These findings speak to the importance of ensuring government regulation of ED advisory statements is in accordance with best practice guidelines and that the labels form part of an integrated multi-faceted broader campaign seeking to educate and inform consumers, so that they are informed of the severity and probability of harm. Given that Australia is considered by industry to have the tightest regulation of the ED market [43], these findings also speak to the need for greater regulatory action internationally to ensure that consumers are to make informed decisions regarding their levels of ED consumption.

## Additional file

Additional file 1:
**Demographic Profile and AmED Consumption Practices of Past Year ED Consumers According to Guideline Awareness.** (Original Groups). (DOCX 15 kb)

## Abbreviations

ED: Energy drink
AmED: Alcohol mixed with energy drink
US: United States
OR: Odds ratio
95 % CI: 95CI Confidence interval
M: Mean
SD: Standard deviation
