# Supplementary material for: Awareness of energy drink intake guidelines and associated consumption practices: a cross-sectional study
Source: BMC Public Health. 2016 Jan 5;16:6. doi: 10.1186/s12889-015-2685-2 (PMC4700652; doi:10.1186/s12889-015-2685-2)
Supplement: Additional file 1: — Demographic Profile and AmED Consumption Practices of Past Year ED Consumers According to Guideline Awareness. (Original Groups). (DOCX 15 kb) [file 12889_2015_2685_MOESM1_ESM.docx]

Additional File 1

*Demographic Profile and AmED Consumption Practices of Past Year ED Consumers According to Guideline Awareness*

*(Original Groups)*

|  | **A.**  **Accurate Estimators**  n=427 | **B.**  **Under-Estimators**  n=350 | **C.**  **Over-Estimators**  n=51 | **D.**  **Unaware Consumers**  n=302 | **B vs A (ref)**  OR (95% CI) | **C vs A (ref)**  OR (95% CI) | **D vs A (ref)**  OR (95% CI) |
| --- | --- | --- | --- | --- | --- | --- | --- |
| **Demographics and other Drug Use** |  |  |  |  |  |  |  |
| Age (M, SD) | 23.5 (5.2) | 24.1 (6.4) | 24.9 (7.6) | 26.0 (8.3) | 1.02 (0.99-1.04), p=.197 | 1.04 (0.99-1.08), p=.108 | **1.06 (1.03-1.08), p<.001** |
| % Male | 53 (48-58) | 61 (55-66) | 47 (34-61) | 63 (57-68) | **1.40 (1.05-1.87), p=.022** | 0.78 (0.44-1.40), p=.409 | **1.49 (1.10-2.01), p=.010** |
| % Employed | 57 (53-62) | 65 (60-70) | 59 (45-71) | 59 (53-64) | **1.37 (1.02-1.84), p=.034** | 1.06 (0.59-1.91), p=.843 | 1.05 (0.78-1.41), p=.768 |
| % Completed tertiary qualification | 46 (41-50) | 48 (43-53) | 53 (40-66) | 49 (42-54) | 1.10 (0.83-1.46), p=.519 | 1.34 (0.75-2.40), p=.325 | 1.12 (0.83-1.51), p=.447 |
| % Currently studying tertiary qualification | 80 (76-84) | 79 (75-83) | 77 (63-86) | 78 (73-82) | 0.93 (0.65-1.32), p=.682 | 0.80 (0.40-1.59), p=.517 | 0.86 (0.60-1.23), p=.410 |
| Daily caffeine intake (exl. ED; M, SD) | 200.5 (199.5) | 192.4 (154.7) | 202.1 (161.5) | 224.5 (213.4) | 1.00 (1.00-1.00), p=.529 | 1.00 (1.00-1.00), p=.956 | 1.00 (1.00-1.00), p=.107 |
| **AmED Use (past year)** | 69 (65-74)  n=296 | 66 (61-71)  n=230 | 63 (49-75)  n=32 | 56 (50-62)  n=169 | 0.85 (0.63-1.15), p=.285 | 0.75 (0.41-1.36), p=.340 | **0.56 (0.41-0.76), p<.001** |
| *Amongst consumers:* |  |  |  |  |  |  |  |
| % Fortnightly+ any AmED use | 25 (20-30) | 17 (13-22) | 16 (7-33) | 13 (9-19) | **0.63 (0.41-0.97), p=.037** | 0.59 (0.22-1.60), p=.302 | **0.47 (0.28-0.79), p=.004** |
| Typical ED intake (M, SD) | 3.0 (2.1) | 3.0 (2.6) | 3.2 (2.4) | 3.2 (3.1) | 1.00 (0.93-1.07), p=.897 | 1.03 (0.90-1.17), p=.701 | 1.03 (0.96-1.10), p=.461 |
| % Typical ED intake exceeds guideline | 44 (39-50) | 39 (33-46) | 47 (31-64) | 41 (34-49) | 0.82 (0.57-1.17), p=.264 | 1.12 (0.54-2.33), p=.764 | 0.88 (0.60-1.30), p=.516 |
| Typical alcohol intake (M, SD) | 6.5 (4.4) | 5.3 (3.6) | 5.5 (3.6) | 5.8 (4.4) | **0.93 (0.89-0.98), p=.002** | 0.94 (0.86-1.04), p=.225 | 0.97 (0.92-1.01), p=.140 |
| % Typical alcohol intake exceeds guideline | 59 (53-65) | 52 (45-58) | 48 (32-65) | 50 (43-58) | *0.74 (0.52-1.04), p=.084* | 0.65 (0.31-1.36), p=.253 | *0.69 (0.47-1.01), p=.059* |
| Maximum ED intake (M, SD) | 4 (2.9) | 3.7 (3.0) | 3.5 (2.0) | 3.7 (3.3) | 0.96 (0.91-1.02), p=.208 | 0.95 (0.83-1.09), p=.435 | 0.97 (0.91-1.04), p=.416 |
| % Maximum ED intake exceeds guideline | 60 (54-65) | 54 (47-60) | 56 (39-72) | 56 (49-64) | 0.78 (0.55-1.11), p=.174 | 0.87 (0.42-1.82), p=.716 | 0.87 (0.59-1.29), p=.492 |
| Maximum alcohol intake (M, SD) | 9.0 (6.6) | 7.7 (6.5) | 8.5 (7.1) | 7.6 (6.8) | **0.97 (0.94-1.00), p=.026** | 0.99 (0.94-1.04), p=.668 | **0.97 (0.94-1.00), p=.027** |
| % Maximum alcohol intake exceeds guideline | 71 (65-76) | 60 (53-66) | 56 (39-72) | 59 (51-66) | 0.78 (0.55-1.11), p=.174 | 0.87 (0.42-1.82), p=.716 | 0.87 (0.59-1.29), p=.492 |

*Note.* Those values bolded indicate statistical significance (*p*<.050) and those italicised indicated a trend towards statistical significance (*p*<.100). ED: energy drink; AmED: alcohol mixed with energy drink; M: mean; SD: standard deviation.
